# Optimization of Emission Reduction Target in the Beijing–Tianjin–Hebei Region: An Atmospheric Transfer Coefficient Matrix Perspective

**DOI:** 10.3390/ijerph192013512

**Published:** 2022-10-19

**Authors:** Yuan Wang, Zhou Pan, Yue Li, Yaling Lu, Yiming Dong, Liying Ping

**Affiliations:** 1 School of Environmental Science and Engineering, Tianjin University, Tianjin 300072, China; 2 Environmental Research Centre of Beijing-Tianjin-Hebei Region, Chinese Academy of Environmental Planning, Beijing 100012, China; 3 School of Environment, Beijing Normal University, Beijing 100091, China

**Keywords:** air pollutants, emission reduction assessment, optimized scenario analysis, WRF/CALPUFF, linear optimization model

## Abstract

In recent years, the problem of atmospheric pollution has been concerning in the Beijing–Tianjin–Hebei region, due to the frequent haze. It has become a significant issue to improve regional air quality through appropriate emission reduction measures. In this study, considering the regional atmospheric transmission of air pollutants, the WRF/CALPUFF model (the Weather Research and Forecasting model coupled with the California Puff air quality model) was used to describe the impact of each city’s pollutant emissions on the concentrations of every city. Then, a new optimization model was designed to calculate the maximum allowable emissions of every city. The results showed that NOx and PM_2.5_ emissions need to be reduced by 44% and 48%, respectively, in the traditional mitigation scenario (any city’s pollutant emissions are not allowed to increase). However, in the optimized scenario, NOx and PM_2.5_ emissions should be reduced by 23% and 46%, respectively, to meet the national secondary standard. The emissions of cities with low transfer coefficients, such as Zhangjiakou, Qinhuangdao, and Chengde, could even be appropriately increased. This means that the optimized scenario could reduce the pressure on emission reduction. Although the optimization results are theoretical and idealistic, this research study provides a new idea for formulating emission mitigation policies in various regions to reduce the impact on the economy.

## 1. Introduction

In recent years, the continual industrialization, urbanization, and regional economic integration in China have led to the problems of compound atmospheric pollution and carbon dioxide (CO_2_) emission [1,2,3,4]. Toxic air pollutants and CO_2_ are the world’s most significant threats to human health, are major contributors to regional inequality and environmental injustice [5,6], and have profound impacts on the functioning of modern human societies [7]. This is particularly prominent in the Beijing–Tianjin–Hebei region [8,9]. Moreover, this region has become one of the key areas for air pollution control in China [10,11]. Therefore, it is urgent for Beijing, Tianjin, and Hebei to formulate reasonable emission reduction targets. However, air quality is not only affected by local emissions, but it is also affected by very significant regional transmission. If the emission reduction targets are set according to the local emissions of each city independently, it may not be the best strategy. 

At present, there are many studies on air pollution transmission across regions or cities [8,12,13,14], most of which are the application of the atmospheric quality model estimating the impact of air pollutants from different sources on the atmospheric environmental quality of the region or cities. Chang et al. [15] used the Community Multiscale Air Quality Model equipped with the Integrated Source Apportionment Model to simulate the contributions from five major emission sectors in 13 cities of the Beijing–Tianjin–Hebei region and 4 surrounding provinces outside this region for the year of 2014. Wang et al. [16] used the WRF/CALPUFF model to study the transmission characteristics of four major air pollutants (SO_2_, NOx, PM_2.5_, and PM_10_) in the Beijing–Tianjin–Hebei urban agglomeration in China in winter, which is the season characterized by the highest levels of pollution. The results showed that the local emissions made the largest contribution (40–60%) for most cities in the Beijing–Tianjin–Hebei region [15,16,17]. However, they did not use an optimization model to calculate the minimum emission reduction ratios under certain air quality standards, nor did they take into account the reduction in emissions in some cities and the possible increase in emissions in other cities, but the air quality of the whole region could still meet the standard.

The transmission coefficient matrix of air pollution between cities varies under different meteorological conditions. However, few researchers have studied how to optimize the emission reduction target of each city in an urban agglomeration using the transfer coefficient matrix of air pollutants based on the atmospheric quality model. Most of the existing optimization studies are based on the optimization of specific measures. For example, Elkamel et al. [18] introduced an interactive optimization methodology for allocating the number and configuration of an Air Quality Monitoring Network (AQMN) in a vast area to identify the impact of multiple pollutants. Turrini [19] implemented and solved a non-linear, multi-objective, multi-pollutant decision problem where the decision variables were the application levels of emission abatement measures making the reduction in energy consumption, end-of-pipe technologies, and fuel switch options possible. In addition, a genetic-algorithm-aided stochastic optimization (GASO) model was developed for supporting regional air quality management under uncertainty to generate solutions that contained a spectrum of potential air pollutant treatment options with risk and cost information [20]. Air pollution is a cross-regional problem, and the transmission of air pollution between different regions or cities largely determines the effectiveness of atmospheric environmental management measures. So, it is necessary to optimize the emission reduction targets from a more macro perspective, to avoid the occurrence of emission reduction targets only considering the individual city scale.

The relationship between environment and economic growth has become a prominent topic in recent decades. Analyzing the economy–environment nexus is significant because most countries attempt to limit environmental deterioration while pursuing economic growth [21]. This is an important aspect of atmospheric environmental management from the perspective of joint prevention and control. For example, the unit pollutant emissions of some source cities have a great impact on the air quality of other cities. This kind of cities should bear greater responsibility for emission reduction. Their emission reduction has a greater contribution to the improvement in the atmospheric environment for all the agglomeration. The emissions of some recipient cities are not large, but they are affected by the surrounding cities. Such cities can take relatively small responsibility for reducing emissions. If emission reduction is regarded as increasing the expenditure, this optimization can reduce the average cost of emission reduction and the impact on economic development. In this way, air quality can be improved with the least economic lost. The most important part of the reduction strategy is the assessment of the maximum allowable emissions of pollutants, which is based on the hypothesis that the concentration of pollutants in each city will reach the national air quality standard. The optimized emission reduction assessment method calculates the maximum allowable emission of pollutants for each city not only based on the local emission but also on other factors. For example, Wang and Pan [22] used the ADMS-Urban atmospheric diffusion model and the linear optimization model to calculate the maximum allowable emissions of SO_2_ and NOx pollutants of one city in China, which proved that the optimization result was reliable and feasible in the emission reduction plan. 

In order to achieve the goal of reducing emissions and improving air quality in the Beijing–Tianjin–Hebei region and to also achieve a greater economic benefit cost ratio, the optimized assessment model to calculate the maximum allowable emissions of two typical pollutants (NOx and PM_2.5_) was established in the Beijing–Tianjin–Hebei region. After years of SO_2_ controlling, the concentration of SO_2_ in mainland China has been far lower than the national air quality secondary standard. At present, the Chinese government has not taken SO_2_ as the key controlled pollutant but has taken PM_2.5_ and its precursors, such as NOx and VOCs, as the focus of China’s air pollution prevention and control. Therefore, the maximum allowable emissions of SO_2_ are not calculated. Moreover, the optimal emission of each city to reach the national secondary standard in the future was predicted, and the impact on the economy was assessed. The specific goals of this study were: (1) to obtain the optimized maximum allowable emissions of NOx and PM_2.5_ in the Beijing–Tianjin–Hebei region, in order to provide suggestions for pollution reduction plans; (2) to assess the impact of optimized results on the GDP in order to compare social and economic benefits and make the best decision.

## 2. Materials and Methods

### 2.1. Study Area

In recent years, the rapid development trend of urban agglomerations in China is becoming increasingly apparent based on regional integration [23]. The top three in China are the Beijing–Tian–Hebei region, the Yangtze River Delta region, and the Pearl River Delta region [24]. The assessment area of this study was the Beijing–Tian–Hebei region, where the air quality is the worst. The location of this region is shown in Figure S1 of Supplementary Information; it includes Beijing (BJ), Tianjin (TJ), and 11 cities in Hebei Province, namely, Baoding (BD), Cangzhou (CZ), Chengde (CD), Handan (HD), Hengshui (HS), Langfang (LF), Qinhuangdao (QHD), Shijiazhuang (SJZ), Tangshan (TS), Xingtai (XT), and Zhangjiakou (ZJK). It has a high population density and rapid economic development. Its population density (514.76 per/km^2^) is more than 3 times that of the whole country (142.1 per/km^2^). Moreover, its total GDP is 1.003 trillion USD, accounting for 10.20% of the total GDP of China (MEPC 2017). 

The air pollution in this region is serious in China [25]. Air pollution is affected by many factors, such as geographic location and wind direction, industry, etc. Firstly, natural factors, such as topography and meteorology, have an impact on the atmospheric environment. The Beijing–Tianjin–Hebei region is surrounded by Taihang Mountains and Yanshan Mountains, which are not conducive to the diffusion of air pollutants. There is a pollution belt along the Taihang Mountains [26,27]. However, weather factors (temperature, humidity, and wind speed) also have an effect [25]. Secondly, smoke and dust emissions produced by industrial and household sources are important factors causing air pollution, which account for 70–90% of the total emissions in the Beijing–Tianjin–Hebei region [28]. The pollutant emission intensity in the Beijing–Tianjin–Hebei region is 3–5 times higher than the national one. Hebei Province contributed the largest volume of smoke and dust emissions in China in 2016 [26]. Thirdly, regional transmission is also an important factor in air pollution. Wang [29] revealed that the cross-city transport between cities inside the Beijing–Tianjin–Hebei region contributed 26–35% of PM_2.5_ as compared with local emissions.

### 2.2. Data Source

The emission inventory used in this paper was obtained from Multi-resolution Emission Inventory for China (MEIC) of Tsinghua University. It has a spatial resolution of 0.25 × 0.25 degrees [17]. The emission data from January 2016 concerning NOx and PM_2.5_ emissions from five sources (Industry, Power, Transportation, Resident, and Agriculture) were selected to establish different emission reduction scenarios.

Since many previous Chinese studies used January to represent winter, the season with the highest air pollution level [30,31], the research time range of this paper was set in January 2016. The Weather Research and Forecasting (WRF)/California Puff (CALPUFF) model was used to simulate pollutant concentrations. The historical meteorological data used by the WRF model were derived from the Final Operational Global Analysis (FNL) data of National Centre for Environmental Prediction (NCEP).

The actual monitored pollutant concentrations of two pollutants (NO_2_ and PM_2.5_) were obtained from 81 air quality monitoring stations in the Beijing–Tianjin–Hebei region (Tables S1–S13 of Supplementary Information). The daily average concentrations of two pollutants in January 2016 from Ministry of Environmental Protection Data Centre http://datacenter.mep.gov.cn (accessed on 5 September 2019) were collected; then, the monthly average concentrations of 13 cities were calculated. The actual monthly average concentrations were compared with the simulated concentrations of two pollutants to verify the accuracy of the CALPUFF model simulation. For NOx, the Romberg Scheme method was used to transform the simulated concentration of NOx to that of NO_2_ for comparing with the actual monitored concentration of NO_2_ [32].

### 2.3. Methods

#### 2.3.1. WRF/CALPUFF Model

In this study, the WRF/CALPUFF model was used to establish the transfer coefficient matrixes of NOx and PM_2.5_, which showed the impact of air pollutant transport between cities. The WRF model is a mesoscale meteorological model developed by National Center for Atmospheric Research (NCAR), the national institute of atmospheric sciences in the United States. It is a limited-area, non-hydrostatic, terrain-following sigma-coordinate model designed to simulate or predict regional weather and climate. This model represents the recent advances in regional climate models that combine the expertise and experience of mesoscale meteorology, and land-surface and climate science developed over the last several decades [33]. The CALPUFF model system, simulating the transport, transformation, and removal of pollutants in the atmospheric environment when the three-dimensional flow field changes, consists of three main parts: the diagnostic wind field model (CALMET), the Gaussian smoke mass diffusion model (CALPUFF), and post-processing software (CALPOST) [34]. Moreover, the WRF model is combined with the CALPUFF model by transferring data of the three-dimensional meteorological field to the CALMET model. The simulation parameters of the WRF/CALPUFF model used in this paper can be found in Tables S14 and S15 of Supplementary Information. Moreover, the calibration and localization of model parameters can make simulated concentrations and monitored concentrations show a strong correlation; its verification process can be found in our other paper [16].

#### 2.3.2. Optimization Model

According to the atmospheric transmission rule, the optimization model was established to calculate the maximum allowable emissions of NOx and PM_2.5_. The calculation process is shown below.

##### Transfer Coefficient Matrixes of Pollutants

Firstly, the transfer coefficient matrix encompassing 13 cities was established using the WRF/CALPUFF model. Then, calculations were made when only city j emitted pollutants in the WRF/CALPUFF model to obtain the average pollutant concentration of all 13 cities. T_ij_ represents the transfer coefficient, which means the influence of per unit pollutant emission of city j on the pollutant concentration of city i; then:

(1) $$T_{\mathrm{ij}}=\frac{C_{\mathrm{ij}}}{Q_{j}}$$

where C_ij_ is the average concentration in city i when only city j emitted pollutants and Q_j_ is the pollutant emission of city j.

Using T_ij_ as a matrix element, a transfer coefficient matrix of this pollutant was constructed within the region:

(2) $$T=\left[\begin{array}{cccc} \frac{C_{11}}{Q_{1}} & \frac{C_{12}}{Q_{2}} & \cdots & \frac{C_{1j}}{Q_{j}}\\ \frac{C_{21}}{Q_{1}} & \frac{C_{22}}{Q_{2}} & \cdots & \frac{C_{2j}}{Q_{j}}\\ \vdots & \vdots & \ddots & \vdots \\ \frac{C_{i1}}{Q_{1}} & \frac{C_{i2}}{Q_{2}} & \cdots & \frac{C_{\mathrm{ij}}}{Q_{j}} \end{array}\right]$$

##### Optimization Model

(3) $$\mathrm{Max}\;Q=\sum_{j=1}^{13}Q_{j}\;s.t.\left\{\begin{array}{c} \mathrm{TQ}\leq C_{\mathrm{is}}-C_{\mathrm{ib}}\\ m\leq Q_{j} \end{array}\right.$$

where Max Q is the maximum allowable emission of the Beijing–Tianjin–Hebei region; ‘$\mathrm{Max}\;Q=\sum_{j=1}^{13}Q_{j}$’ means that the maximum allowable emission in this region should not be higher than the sum of current emissions of 13 cities in this region; TQ represents the target concentration of air pollutants in city j; C_is_ is the national air quality secondary standard; C_ib_ is the background concentration of pollutants in city j; ‘$\mathrm{TQ}\leq C_{\mathrm{is}}-C_{\mathrm{ib}}$’ means that the predicted target concentration of pollutants in city j should not exceed the national air quality secondary standard after superposing the background concentration. Moreover, the lower limit (m) for the maximum allowable emissions of pollutants was set. m is the pollutant emission from residents and transportation. This study tried to mainly adjust the pollutant emission from industry and power, which are sectors where the environmental protection department can effectively implement pollution reduction policies, and also tried to minimize the impact of pollution reduction on people’s lives. ‘$m\leq Q_{j}$’ means that the total pollutant emission in city j should be greater than that of residents and transportation. j=1,2,⋯,13 . j represents one of cities in the Beijing–Tianjin–Hebei region. i represents one kind of air pollutants, such as NOx or PM_2.5_.

#### 2.3.3. Comparative Analysis Method of Optimization Results

To evaluate the optimization results, three scenarios for comparison were set up. 

The S0 scenario was the actual emission assessment scenario, as an original control scenario. The emissions of pollutants in S0 were from five sources (Industry, Power, Transportation, Resident, and Agriculture) of the MEIC emission inventory in January 2016. 

The S1 scenario was the emission reduction ratio scenario from the government. For reaching the national air quality secondary standard, based on the principle of anti-degradation of air quality (any city’s pollutant emissions are not allowed to increase), the government calculated the emission reduction ratio of 13 cities (see Supplementary Information Table S21). 

The S2 scenario was the optimization result scenario of this study. This study tried to allow emissions to increase in some cities and obtain the pollutants maximum allowable emission of every city in the Beijing–Tianjin–Hebei region considering the transmission of air pollution between cities (i.e., transfer coefficient matrix T_ij_).

The variation ratio of the maximum allowable emissions, emission intensity, and GDP were calculated under S1 and S2. The calculation formula for variation ratio is as follows:

(4) $$\mathrm{Variation}\;\mathrm{ratio}=\frac{R_{1\mathrm{or}2}-R_{0}}{R_{0}}$$

where R_1or2_ is the maximum allowable emissions, emission intensity, or GDP under S1 or S2; R_0_ is the maximum allowable emissions, emission intensity, or GDP under the actual scenario, S0.

#### 2.3.4. Calculation Method of GDP after Emission Reduction

Here, emission intensity, the number of emissions per unit of GDP, was used as a coefficient linking pollution emissions and the economy. The GDP in S0 was derived from the statistical yearbook of China [35]. The calculation method of GDP of S1 or S2 is shown as follows:

(5) $${\mathrm{GDP}}_{1\mathrm{or}2}=\frac{{\mathrm{APE}}_{1\mathrm{or}2}}{e_{0}}$$

where APE_1or2_ is the atmospheric pollutant equivalent under S1 or S2; e_0_ is the emission intensity under the actual scenario, S0.

The purpose of introducing APE here was to combine the emissions of two pollutants (NOx and PM_2.5_) into one indicator for GDP calculation. APE is a combined equivalent of atmospheric pollutants. Based on China’s pollution charge schedule in China Environmental Protection Tax Law (Ministry of Ecology and Environment of the People’s Republic of China, 2017), the respective conversion coefficients to APE for NOx and Dust are 0.95 and 4, which means that 1 kg of APE is equal to 0.95 and 4 kg of NOx and Dust, respectively. This integration method has shown to be reasonable and feasible for the research of air pollution embodied in trade [14,36].

The calculation formula of APE is:

(6) $$\mathrm{APE}=\frac{E_{\left({\mathrm{NO}}_{x}\right)}}{0.95}+\frac{E_{\left({\mathrm{PM}}_{2.5}\right)}}{4}$$

where E_(NOx)_ and E_(PM2.5)_ are the emissions of NOx and PM_2.5_.

The calculation formula of e_0_ is:

(7) $$e_{0}=\frac{{\mathrm{APE}}_{0}}{{\mathrm{GDP}}_{0}}$$

where APE_0_ and GDP_0_ are APE and GDP under the actual scenario, S0.

### 2.4. Assumptions of This Research Study

**Figure 1:**
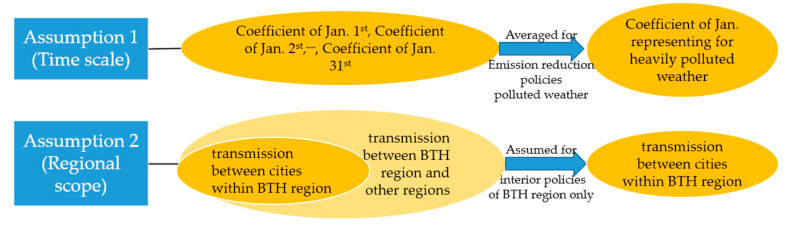
Interpretation of the three assumptions in this study.

This study took the Beijing–Tianjin–Hebei region as a whole, with the goal of obtaining the air environment management measures with the best economic and environmental benefits for each city. The below assumptions (Figure 1) were made.

(1) Assumption 1 is about the time scale of this study. We chose the most severe season in one of the most polluted years (i.e., January 2016) in the Beijing–Tianjin–Hebei region. This assumption was mainly used in the WRF/CALPUFF model and the optimization model (Section 3.1, Section 3.2 and Section 3.3). The transmission coefficient of each city constantly changes, depending on natural conditions such as topography and meteorology, for the relationship between pollutant emission and concentration is affected by many complex physical and chemical mechanisms. However, this study mainly analyzed the optimization of emission reduction responsibility from the perspective of management and took the average results of the transmission coefficient of each city as the optimization parameters.
(2) Assumption 2 is about the scope of the study area. We only considered emissions from the Beijing–Tianjin–Hebei region, excluding other regions. It was mainly used in the WRF/CALPUFF model setup (Section 3.1 and Section 3.2). Although the surrounding areas have a certain impact on the atmospheric environmental quality of Beijing, Tianjin, and Hebei, the impact of cities inside the Beijing–Tianjin–Hebei region is greater. Moreover, when making policies, the Chinese government often considers the Beijing–Tianjin–Hebei region as a whole, without considering the surrounding provinces. Therefore, the results of the interaction between Beijing–Tianjin–Hebei region and its surrounding areas were not the main basis for formulating the emission responsibility of the 13 cities in this paper.

## 3. Results

### 3.1. Validation of the WRF/CALPUFF Model

**Figure 2:**
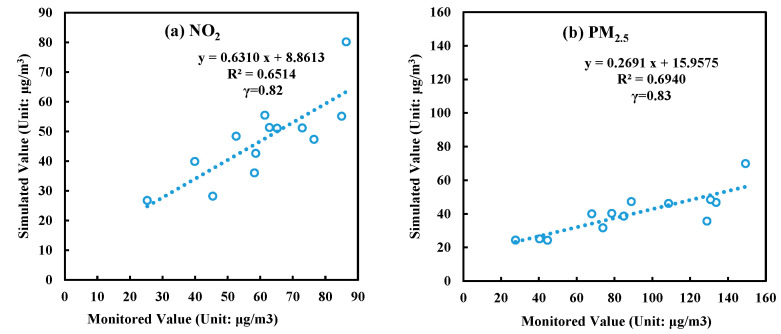
Comparison between simulated values (scenario S0) and monitored values.

The simulated concentrations of NO_2_ and PM_2.5_ in the actual emission assessment scenario (S0) were compared with the monitored concentrations to verify the accuracy of the CALPUFF model simulation. As shown in Figure 2, the correlation coefficients (γ) between the simulated and monitored values of NO_2_ and PM_2.5_ were 0.81 and 0.83, respectively. The correlation coefficient is an indicator to measure the degree of correlation between simulated and monitored values. Generally, the larger the correlation coefficient is, the higher the degree of correlation is. The respective goodness-of-fit values of their regression line (R^2^), which is used to test the regression models and compare the simulated results with monitored values, were 0.65 and 0.69. The simulated concentration and actual monitored concentration were significantly correlated at the 0.01 level (both sides). The analysis showed that the CALPUFF air quality model and simulation parameters used in this paper could be used to analyze the relationship between emission and concentration.

### 3.2. Pollutant Transfer Coefficients

Cities with larger transfer coefficients have greater negative impacts on themselves and other cities. Therefore, it was considered that these cities implemented a larger emission reduction ratio. Cities with smaller transfer coefficients have less impacts on themselves and other cities in terms of pollutant emissions. Therefore, we considered that these cities could implement smaller pollutant emission reduction rates. Judging from the overall reduction in emissions and the improvement in air quality in the Beijing–Tianjin–Hebei region, the emissions of cities with smaller transfer coefficients could even be appropriately increased to a certain extent, because the environmental benefits brought by the reduction in emissions in cities with large transfer coefficients could compensate for the environmental losses caused by the increase in emissions from cities with small transfer coefficients. At the same time, the economic benefits brought about by the increase in emissions in cities with a small transfer coefficient could also make up for the economic losses caused by the reduction in emissions in cities with a large transfer coefficient. In this way, not only could the air quality in this region be improved, but economic losses could also be reduced.

The transmission matrixes can be seen in Tables S16 and S17 of Supplementary Information. Table S16 shows the NOx transfer coefficient among cities in the Beijing–Tianjin–Hebei region. Cities with large NOx transfer coefficients included Shijiazhuang, Handan, and Baoding, and their transfer coefficients were greater than 0.0089 μg/(m^3^·t). Therefore, we considered these cities to implement a larger proportion of NOx reduction. On the other hand, cities with low NOx transfer coefficients included Zhangjiakou, Qinhuangdao, and Chengde, and their transfer coefficients were less than 0.0059 μg/(m^3^·t). Therefore, it was considered that these cities reduced NOx emissions in a smaller proportion. On the premise that the local air quality still met the standard, the NOx emissions of these cities could even be appropriately increased. Table S17 shows a similar pattern.

### 3.3. Maximum Allowable Emissions

**Figure 3:**
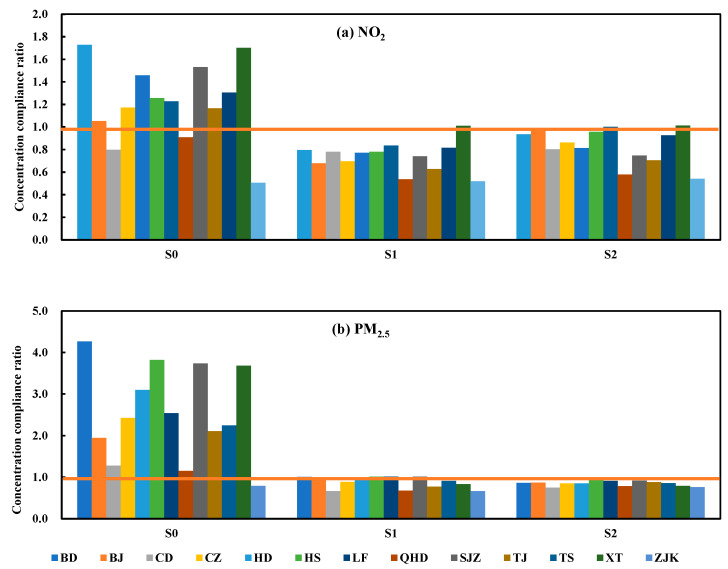
Concentration compliance ratios of two pollutants under different emission scenarios for every city (the orange line is the national air quality secondary standard; the concentration compliance ratio is equal to the concentration value of each city divided by the national secondary standard value).

**Figure 4:**
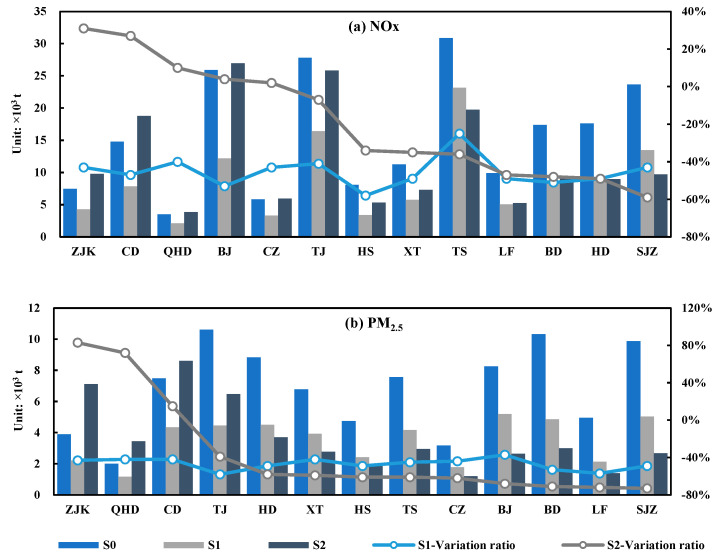
Emissions of two pollutants (NOx and PM_2.5_) under different emission scenarios.

The maximum allowable emissions of pollutants under the two emission reduction scenarios were put into the CALPUFF model to simulate the concentration of pollutants. The results showed that the concentration of NOx and PM_2.5_ under the two emission reduction scenarios could meet the national air quality secondary standard (Figure 3). The variation ratio of emissions under the two emission reduction scenarios was assessed using Equation (1) (see Figure 4 and Supplementary Information Tables S18 and S19). The maximum allowable emissions of two pollutants (NOx and PM_2.5_) in scenario S1 were 114,330.72 t and 46 186.69 t, respectively. Compared with the actual emission scenario (S0), the emission variation ratio of NOx in scenario S1 was −44%, and the emission variation ratio of PM_2.5_ was −48%. The maximum allowable emissions of two pollutants (NOx and PM_2.5_) in the optimized emission reduction scenario (S2) were 156,407.33 t and 47,824.49 t, respectively. Compared with the actual emission scenario (S0), the average variation ratios of two pollutants (NOx and PM_2.5_) were −23% and −46%, respectively.

From the results of maximum allowable emissions, the emission reduction ratios of two pollutants in S1 (44% and 48%, respectively) were significantly greater than those in S2 (23% and 46%, respectively). In the optimized emission reduction scenario (S2), emissions could increase in some cities; taking PM_2.5_ as an example, the transfer coefficient of cities with the largest transfer coefficient (e.g., Shijiazhuang) could reach more than 0.20 μg/m^3^·t, and these cities needed to reduce emissions by more than 60%. Moreover, the transfer coefficient of cities with small transfer coefficients (e.g., Zhangjiakou) was below 0.03 μg/m^3^·t, and the emissions of these cities could increase, because these cities not only had low transfer coefficients, but they also had low local pollutant concentrations. Therefore, even if they increased their emissions, they would have little impact on the surrounding area, and the local pollutant concentration would not exceed the standard. The difference in the transfer contribution of each city could provide the conditions for optimizing urban emission reduction targets.

### 3.4. Impact of Emission Changes on Economy (GDP)

The specific GDP and the variation ratio of GDP in each city under the two emission reduction scenarios can be found in Supplementary Information Table S19.

**Figure 5:**
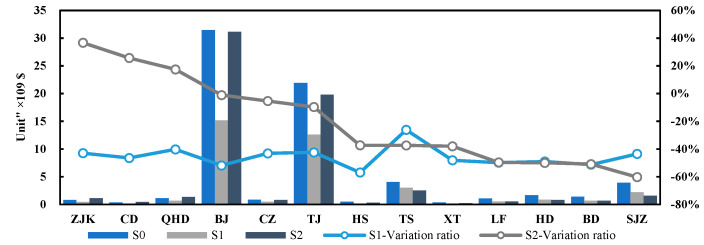
GDP of two pollutants (NOx and PM_2.5_) under different emission scenarios.

In scenario S1, the average GDP in the Beijing–Tianjin–Hebei region was 2.87 trillion USD. Compared with the actual GDP, the variation ratio of GDP in scenario S1 was about −46% (Figure 5). In the optimized emission reduction scenario S2, the average GDP of the Beijing–Tianjin–Hebei region was 4.72 trillion USD, and the average variation ratio of GDP was only about −12% (Figure 5). For the emissions of pollutants in cities with smaller transfer coefficients (Zhangjiakou, Chengde, etc.), there was still environmental capacity for emissions in these cities. The GDP of these cities could increase accordingly. Obviously, the emission reduction in pollutants under the optimized emission reduction scenario (S2) had the least impact on the overall economy of the Beijing–Tianjin–Hebei region due to the smaller total emission reduction.

### 3.5. Impact of Emission Intensity Changes on GDP

Section 3.4 above reports the variation ratio of the GDP when the emission intensity remained unchanged. According to the statistical emission and GDP data from 2012 to 2016, the average annual emission intensity of major air pollutants decreased by about 10–20%. If we considered the reduction in emission intensity, the negative impact of emission reduction on the GDP would be reduced.

Therefore, we set the following three cases: In S1 and S2, the emission intensity of APE was unchanged. In S1′ and S2′, the emission intensity of APE was reduced by 20%. In S1″ and S2″, the emission intensity of APE was reduced by 50%. We then compared the variation ratio of GDP in these three cases, respectively.

**Figure 6:**
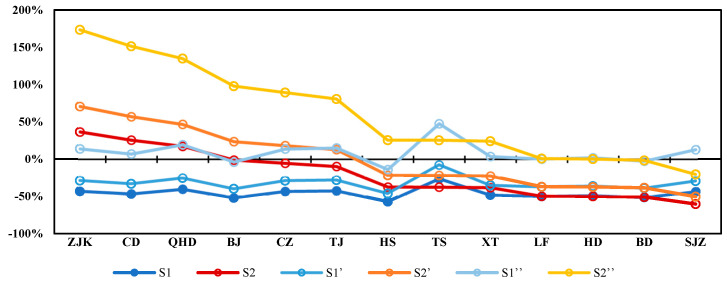
GDP variation ratios under different emission and emission intensity scenarios (S1 and S2, unchanged emission intensity; S1′ and S2′, emission intensity reduced by 20%, S1″ and S2″, emission intensity reduced by 50%).

In S1, S1′, and S1″, the average GDP variation ratios of the 13 cities in Beijing–Tianjin–Hebei were −46%, −32%, and 9%, respectively. It showed that in the scenario S1, only a 50% reduction in emission intensity could compensate for the GDP loss caused by pollutant emission reduction (Figure 6). However, relying on technological innovation to reduce emission intensity by 50% would be difficult to achieve in the short term.

In S2, S2′, and S2″, the average GDP variation ratios of the 13 cities in Beijing–Tianjin–Hebei were −20%, 0%, and 60%, respectively. It showed that in the optimized emission reduction scenario (S2), only a 20% reduction in emission intensity was needed to make up for the GDP loss caused by pollutant emission reduction. Therefore, the S2 emission reduction plan would be more economical.

### 3.6. Discussion

In this paper, the optimized emission reduction targets could mitigate the impact on the economy as much as possible. The optimized scenario result, i.e., scenario S2, showed that the emissions of NOx should only be reduced by 23%, and the impact on GDP could also be halved compared with scenario S1. If combined with the effect of emission intensity reduction brought about by technological progress, GDP could even show no loss nor increase. For example, if the intensity of emissions were reduced by 20%, there would be no loss of GDP based on the optimized emission reduction results of this paper. According to the data from 2012 to 2016, the average annual emission intensity of major air pollutants decreased by about 10–20%. In the future, the emission intensity of NOx and PM_2.5_ is likely to reach about 20%, which means that the atmospheric environmental quality in the Beijing–Tianjin–Hebei region could reach the secondary standard under the condition of minimizing economic losses.

## 4. Conclusions and Suggestions

### 4.1. Conclusions

This paper used the contribution discrepancy in each city’s emissions to the overall atmospheric environmental quality of the Beijing–Tianjin–Hebei region to carry out the optimization design of the maximum allowable emissions. Then, it assessed the impact of optimized results on the GDP in order to compare social and economic benefits.

(1) For those cities with smaller transfer coefficients, such as Zhangjiakou (with a transfer coefficient of 0.64 × 10^−3^ μg/m^3^·t to Baoding), even if the emission of these cities increased, the impact on the local and surrounding atmospheric environment quality would be relatively small; for cities with larger transfer coefficients, such as Shijiazhuang (with a transfer coefficient of 49.74 × 10^−3^ μg/m^3^·t to Baoding), reducing emissions would not only be beneficial to the local atmospheric environment quality, but it could also improve the atmospheric environment quality of other cities and reduce the pressure of emission reduction in other cities. It is also an important aspect of joint air defense and control work to make full use of the emission reduction optimization brought by atmospheric transmission.
(2) This study tried to provide a new perspective of regional air pollution prevention and control, that is, to optimize emission reduction targets according to the natural spatial differences between emissions and atmospheric environmental quality. If cities adopted the traditional and unified emission reduction ratio policy (scenario S1), the maximum allowable emissions of NOx and PM_2.5_ in the Beijing–Tianjin–Hebei region would be 114,330.72 t and 46,186.69 t, respectively. Considering the regional air pollution transmission and joint prevention and control with the optimization method (scenario S2), the maximum allowable emissions of NOx and PM_2.5_ in this region would be 156,407.33 t and 47,824.49 t, respectively. The concentration of NOx and PM_2.5_ under these two scenarios could both meet the national air quality secondary standard, but the latter is easier and more economical.
(3) In general, the reduction measures of pollutants would lead to the deceleration or loss of the economy. However, from the perspective of regional air pollution joint prevention and control, and economic development, emissions cannot be required to fall in all cities. On the premise of insisting on improving emission reduction technology, we assessed the economic impact of pollutant reduction, optimized the emission reduction proportion of each city instead of simply setting the emission reduction ratio based on the principle of anti-degradation of air quality (any city’s pollutant emissions are not allowed to increase), and reduced the negative impact of pollution reduction on the economy. In scenario S1, the average GDP in the Beijing–Tianjin–Hebei region was 2.87 USD USD. In scenario S2, the average GDP of the Beijing–Tianjin–Hebei region was 4.72 USD USD, which is much higher than that of scenario S1.

### 4.2. Suggestions

(1) When making local policies, it is suggested that policy makers allow cities with a great impact on regional air quality to reduce emissions more and allow cities with a small impact on regional air quality to pursue less emission reduction or even increase emissions. In this way, the overall pressure of emission reduction would be reduced, and the impact on the economy would also be mitigated.
(2) Local governments are advised to improve air quality without affecting economic development by reducing pollutant emission intensity. They are suggested to adjust the industrial structure, eliminate backward enterprises, control the amount of coal consumption, find clean and efficient energy varieties, and seek better energy management methods.
(3) Pollutant emission reduction can depend not only on the reduction in industrial source emissions, but also on the reduction in the emissions of residents and traffic sources. Residents are advised to use clean heating and reduce the amount of coal burning in winter. In order to reduce the emissions of traffic source pollutants, the proportion of electric vehicles should be increased.

In addition, this study discussed an optimization method based on the differences in the impact of urban pollutants emissions on the concentration, which could achieve higher emission reduction benefits and social benefits. However, it also had some limitations. This article set some assumptions about the transfer coefficient. In fact, it is affected by many complex physical and chemical mechanisms. The change in meteorological conditions and pollutant emissions leads to the uncertainty change in this indicator. This study mainly analyzed the optimization of emission reduction responsibility from the perspective of management and took the results of physical and chemical mechanisms as optimization parameters. This limitation could be overcome by further studies of atmospheric models and mechanisms in the future. Moreover, fine research under different meteorological conditions could be carried out to put forward more refined management suggestions.

## Data Availability

The data are not publicly available, and any inquiries may be addressed by the first author.

## Supplementary Materials

The following supporting information can be downloaded at: https://www.mdpi.com/article/10.3390/ijerph192013512/s1, Figure S1: Domain coverage, Table S1: Concentrations monitored by air quality monitoring stations in Beijing, Table S2: Concentrations monitored by air quality monitoring stations in Tianjin, Table S3: Concentrations monitored by air quality monitoring stations in Hengshui, Table S4: Concentrations monitored by air quality monitoring stations in Baoding, Table S5: Concentrations monitored by air quality monitoring stations in Cangzhou, Table S6: Concentrations monitored by air quality monitoring stations in Chengde, Table S7: Concentrations monitored by air quality monitoring stations in Handan, Table S8: Concentrations monitored by air quality monitoring stations in Langfang, Table S9: Concentrations monitored by air quality monitoring stations in Qinhuangdao, Table S10: Concentrations monitored by air quality monitoring stations in Shijiazhuang, Table S11: Concentrations monitored by air quality monitoring stations in Tangshan, Table S12: Concentrations monitored by air quality monitoring stations in Xingtai, Table S13: Concentrations monitored by air quality monitoring stations in Zhangjiakou, Table S14: Parameterization scheme for WRF, Table S15: Parameterization used in CALPUFF, Table S16: The transfer coefficient matrix of NO_x_, Table S17: The transfer coefficient matrix of PM_2.5_, Table S18: The maximum allowable emissions and the emissions variation ratio of three pollutants under different emission scenarios, Table S19: The GDP and the variation ratio of GDP under different emission scenarios, Table S20: The variation ratio of GDP under three hypothetical cases, Table S21: Emission reduction ratio in scenario S1 (relative to 2016).
