# Supplementary material for: Optimization of Emission Reduction Target in the Beijing–Tianjin–Hebei Region: An Atmospheric Transfer Coefficient Matrix Perspective
Source: Int J Environ Res Public Health. 2022 Oct 19;19(20):13512. doi: 10.3390/ijerph192013512 (PMC9603400; doi:10.3390/ijerph192013512)
Supplement: Supplementary file 1 [file ijerph-19-13512-s001.zip › ijerph-1916323-supplementary.pdf]

International Journal of Environmental Research and Public Health

Supplementary Information for *"Optimization of Emission Reduction Target in Beijing–Tianjin–Hebei Region: An Atmospheric transfer coefficient matrix perspective"*

Yuan Wang [1], Zhou Pan [1], Yue Li[1], Yaling Lu [1,2,]*, Yiming Dong[3], and Liying Ping[1]*

[1] School of Environmental Science and Engineering, Tianjin University, Tianjin 300072, China
[2] Environmental Research Centre of Beijing-Tianjin-Hebei Region, Chinese Academy of Environmental Planning, Beijing 100012, China
[3] School of Environment, Beijing Normal University, Beijing 100091, China
* Correspondence: luyl@caep.org.cn (Y.L.); pingly45_@tju.edu.cn (L.P.)

There are 21 Tables and 1 Figure.

Table S1. Concentrations monitored by air quality monitoring stations in Beijing.

City	Air Quality Monitoring Station	Date	Monitored Concentrations (Unit: μg/m^3)	
			NO_2	$PM_{2.5}$
Beijing	1. Wanshou Temple<br>2. West Park officials<br>3. Changping Dingling<br>4. Dongsi<br>5. Temple of Heaven<br>6. Shunyi New Town<br>7. Changping Town<br>8. Chaoyang Agricultural Exhibition Hall<br>9. Huairou town<br>10. Haidian Wanliu<br>11. Shijingshan city<br>12. Chaoyang Olympic Sports Center	2016/1/1	106	176
		2016/1/2	124	266
		2016/1/3	83	247
		2016/1/4	33	37
		2016/1/5	38	34
		2016/1/6	35	25
		2016/1/7	28	13
		2016/1/8	43	35
		2016/1/9	74	76
		2016/1/10	55	46
		2016/1/11	25	11
		2016/1/12	30	14
		2016/1/13	49	26
		2016/1/14	73	73
		2016/1/15	84	100
		2016/1/16	71	123
		2016/1/17	29	37
		2016/1/18	18	10
		2016/1/19	62	62
		2016/1/20	78	117
		2016/1/21	68	131
		2016/1/22	16	13
		2016/1/23	12	9
		2016/1/24	24	13
		2016/1/25	43	28
		2016/1/26	37	35
		2016/1/27	79	98
		2016/1/28	80	121
		2016/1/29	55	62
		2016/1/30	48	50
		2016/1/31	30	18
**Average concentrations**			**52.6**	**67.94**

Table S2. Concentrations monitored by air quality monitoring stations in Tianjin.

City	Air Quality Monitoring Station	Date	Monitored Concentrations (Unit: μg/m^3)	
			NO_2	$PM_{2.5}$
		2016/1/1	79	176
		2016/1/2	137	334
		2016/1/3	53	94
		2016/1/4	63	104
		2016/1/5	45	37
		2016/1/6	51	45
		2016/1/7	36	26
		2016/1/8	45	30
	1. Qinjian street	2016/1/9	98	188
	2. Dazhigu No.8 road	2016/1/10	70	117
	3. Qianjin street	2016/1/11	44	27
	4. Huaihe street	2016/1/12	41	28
	5. Yuejin Road	2016/1/13	73	72
	6. No.4 street	2016/1/14	101	104
	7. Yongming road	2016/1/15	107	175
Tianjin	8. Hanbei road	2016/1/16	46	82
	9. Tuanpowa	2016/1/17	46	60
	10. Hexi Yijing road	2016/1/18	29	23
	11. Jingu Road	2016/1/19	58	39
	12. Binshui Xidao	2016/1/20	70	61
	13. Dali Road	2016/1/21	50	54
	14. Zhongshan North Road	2016/1/22	24	25
	15. Xisi Road	2016/1/23	14	11
		2016/1/24	28	21
		2016/1/25	56	47
		2016/1/26	52	39
		2016/1/27	82	80
		2016/1/28	77	88
		2016/1/29	36	22
		2016/1/30	55	40
		2016/1/31	39	39
**Average concentrations**			**58.2**	**73.81**

Table S3. Concentrations monitored by air quality monitoring stations in Hengshui.

City	Air Quality Monitoring Station	Date	Monitored Concentrations (Unit: μg/m^3)	
			NO_2	$PM_{2.5}$
Hengshui	1. Hengshui City Environmental Protection Bureau<br>2. Electrical Machine North Factory<br>3. Environmental Monitoring Station	2016/1/1	61	171
		2016/1/2	105	375
		2016/1/3	83	380
		2016/1/4	57	135
		2016/1/5	45	58
		2016/1/6	64	121
		2016/1/7	48	69
		2016/1/8	80	179
		2016/1/9	82	248
		2016/1/10	76	276
		2016/1/11	48	53
		2016/1/12	60	98
		2016/1/13	76	129
		2016/1/14	73	123
		2016/1/15	90	168
		2016/1/16	77	204
		2016/1/17	45	95
		2016/1/18	57	122
		2016/1/19	58	81
		2016/1/20	70	91
		2016/1/21	73	132
		2016/1/22	39	56
		2016/1/23	26	37
		2016/1/24	26	39
		2016/1/25	55	89
		2016/1/26	61	83
		2016/1/27	73	106
		2016/1/28	98	170
		2016/1/29	37	60
		2016/1/30	38	53
		2016/1/31	66	143
**Average concentrations**			**62.8**	**133.68**

Table S4. Concentrations monitored by air quality monitoring stations in Baoding.

City	Air Quality Monitoring Station	Date	Monitored Concentrations (Unit: μg/m^3)	
			NO_2	$PM_{2.5}$
Baoding	1. Huadian II<br>2. Reception Center<br>3. City Monitoring Station<br>4. Natatorium<br>5. Film Factory<br>6. Surface water Company	2016/1/1	149	243
		2016/1/2	180	395
		2016/1/3	130	282
		2016/1/4	72	154
		2016/1/5	66	128
		2016/1/6	90	186
		2016/1/7	68	114
		2016/1/8	87	182
		2016/1/9	116	244
		2016/1/10	88	145
		2016/1/11	56	78
		2016/1/12	78	160
		2016/1/13	101	209
		2016/1/14	102	178
		2016/1/15	117	210
		2016/1/16	86	153
		2016/1/17	62	87
		2016/1/18	62	85
		2016/1/19	84	123
		2016/1/20	112	230
		2016/1/21	82	124
		2016/1/22	35	41
		2016/1/23	25	34
		2016/1/24	38	31
		2016/1/25	91	143
		2016/1/26	86	104
		2016/1/27	118	204
		2016/1/28	112	126
		2016/1/29	64	60
		2016/1/30	53	58
		2016/1/31	67	116
**Average concentrations**			**86.4**	**149.26**

Table S5. Concentrations monitored by air quality monitoring stations in Cangzhou.

City	Air Quality Monitoring Station	Date	Monitored Concentrations (Unit: μg/m³)	
			NO_2	$PM_{2.5}$
Cangzhou	1. Municipal Environmental Protection Agency<br>2. TV Broadcast Station<br>3. Cangxian Urban Construction Bureau	2016/1/1	69	167
		2016/1/2	104	296
		2016/1/3	60	135
		2016/1/4	81	90
		2016/1/5	57	50
		2016/1/6	74	79
		2016/1/7	46	32
		2016/1/8	64	73
		2016/1/9	95	186
		2016/1/10	73	161
		2016/1/11	49	34
		2016/1/12	54	58
		2016/1/13	86	127
		2016/1/14	103	179
		2016/1/15	109	177
		2016/1/16	70	99
		2016/1/17	57	76
		2016/1/18	54	57
		2016/1/19	68	51
		2016/1/20	70	61
		2016/1/21	55	64
		2016/1/22	25	31
		2016/1/23	19	23
		2016/1/24	26	24
		2016/1/25	43	49
		2016/1/26	38	39
		2016/1/27	41	33
		2016/1/28	44	67
		2016/1/29	20	29
		2016/1/30	29	40
		2016/1/31	34	43
**Average concentrations**			**58.6**	**84.84**

Table S6. Concentrations monitored by air quality monitoring stations in Chengde.

City	Air Quality Monitoring Station	Date	Monitored Concentrations (Unit: μg/m^3)	
			NO_2	$PM_{2.5}$
Chengde	1. Ligong<br>2. Bank of China<br>3. Economic and Technological Development Zone<br>4. Cultural Center	2016/1/1	69	107
		2016/1/2	87	149
		2016/1/3	81	130
		2016/1/4	20	19
		2016/1/5	36	20
		2016/1/6	28	26
		2016/1/7	18	17
		2016/1/8	35	29
		2016/1/9	54	50
		2016/1/10	36	41
		2016/1/11	22	18
		2016/1/12	24	19
		2016/1/13	35	27
		2016/1/14	56	48
		2016/1/15	58	56
		2016/1/16	37	65
		2016/1/17	30	53
		2016/1/18	15	19
		2016/1/19	44	42
		2016/1/20	57	44
		2016/1/21	50	50
		2016/1/22	11	18
		2016/1/23	6	17
		2016/1/24	10	16
		2016/1/25	36	30
		2016/1/26	48	41
		2016/1/27	60	54
		2016/1/28	64	70
		2016/1/29	51	46
		2016/1/30	32	36
		2016/1/31	28	25
**Average concentrations**			**39.9**	**44.58**

Table S7. Concentrations monitored by air quality monitoring stations in Handan.

City	Air Quality Monitoring Station	Date	Monitored Concentrations (Unit: μg/m^3)	
			NO_2	$PM_{2.5}$
Handan	1. East Sewage Factory<br>2. Municipal Environmental Protection Agency<br>3. CongTai Park<br>4. Mine Hospital	2016/1/1	78	122
		2016/1/2	153	332
		2016/1/3	152	268
		2016/1/4	74	84
		2016/1/5	66	74
		2016/1/6	65	93
		2016/1/7	71	140
		2016/1/8	80	152
		2016/1/9	68	158
		2016/1/10	93	220
		2016/1/11	53	45
		2016/1/12	56	54
		2016/1/13	76	114
		2016/1/14	68	60
		2016/1/15	78	100
		2016/1/16	94	162
		2016/1/17	52	91
		2016/1/18	51	70
		2016/1/19	77	112
		2016/1/20	76	92
		2016/1/21	91	153
		2016/1/22	49	58
		2016/1/23	34	34
		2016/1/24	46	32
		2016/1/25	59	46
		2016/1/26	58	37
		2016/1/27	83	125
		2016/1/28	96	130
		2016/1/29	64	90
		2016/1/30	41	43
		2016/1/31	57	74
**Average concentrations**			**72.9**	**108.55**

Table S8. Concentrations monitored by air quality monitoring stations in Langfang.

City	Air Quality Monitoring Station	Date	Monitored Concentrations (Unit: μg/m^3)	
			NO_2	$PM_{2.5}$
Langfang	1. Environmental Monitoring Supervision Center<br>2. TCM Medical Material Company<br>3. Economic and Technological Development Zone<br>4. Hebei University of Technology<br>5. North China Institute of Aerospace	2016/1/1	115	287
		2016/1/2	137	350
		2016/1/3	55	160
		2016/1/4	54	79
		2016/1/5	45	41
		2016/1/6	46	43
		2016/1/7	34	21
		2016/1/8	39	23
		2016/1/9	103	186
		2016/1/10	73	136
		2016/1/11	35	21
		2016/1/12	45	35
		2016/1/13	71	76
		2016/1/14	92	153
		2016/1/15	97	169
		2016/1/16	55	89
		2016/1/17	32	30
		2016/1/18	22	16
		2016/1/19	55	54
		2016/1/20	86	127
		2016/1/21	59	78
		2016/1/22	28	23
		2016/1/23	24	13
		2016/1/24	39	20
		2016/1/25	72	64
		2016/1/26	48	39
		2016/1/27	96	160
		2016/1/28	93	112
		2016/1/29	51	42
		2016/1/30	62	66
		2016/1/31	40	42
**Average concentrations**			**61.4**	**88.87**

Table S9. Concentrations monitored by air quality monitoring stations in Qinhuangdao.

City	Air Quality Monitoring Station	Date	Monitored Concentrations (Unit: μg/m^3)	
			NO_2	$PM_{2.5}$
Qinhuangdao	1. City Monitoring Station<br>2. Shanhai Pass<br>3. City government<br>4. Beidaihe Environmental Protection Bureau<br>5. Contruction Plazz<br>6. Wenming District	2016/1/1	98	137
		2016/1/2	80	134
		2016/1/3	44	42
		2016/1/4	54	56
		2016/1/5	43	21
		2016/1/6	45	34
		2016/1/7	47	31
		2016/1/8	44	22
		2016/1/9	57	39
		2016/1/10	24	18
		2016/1/11	36	25
		2016/1/12	46	35
		2016/1/13	40	24
		2016/1/14	45	28
		2016/1/15	45	60
		2016/1/16	24	41
		2016/1/17	28	35
		2016/1/18	31	22
		2016/1/19	36	20
		2016/1/20	37	29
		2016/1/21	41	40
		2016/1/22	32	33
		2016/1/23	18	12
		2016/1/24	31	14
		2016/1/25	62	34
		2016/1/26	57	38
		2016/1/27	87	79
		2016/1/28	69	73
		2016/1/29	22	14
		2016/1/30	33	21
		2016/1/31	52	39
**Average concentrations**			**45.4**	**40.32**

Table S10. Concentrations monitored by air quality monitoring stations in Shijiazhuang.

City	Air Quality Monitoring Station	Date	Monitored Concentrations (Unit: μg/m³)	
			NO_2	$PM_{2.5}$
Shijiazhuang	1. Chemical College<br>2. Worker hospital<br>3. High-tech zone<br>4. Northwest Water Company<br>5. Fenglong Montain<br>6. People's Hall<br>7. 22 Central South Campus<br>8. Shiji Park<br>9. Xi'nan College	2016/1/1	122	283
		2016/1/2	169	474
		2016/1/3	139	336
		2016/1/4	88	148
		2016/1/5	67	75
		2016/1/6	85	138
		2016/1/7	69	109
		2016/1/8	66	88
		2016/1/9	94	202
		2016/1/10	95	206
		2016/1/11	46	49
		2016/1/12	67	105
		2016/1/13	77	88
		2016/1/14	61	43
		2016/1/15	92	120
		2016/1/16	86	181
		2016/1/17	58	112
		2016/1/18	44	53
		2016/1/19	81	128
		2016/1/20	91	181
		2016/1/21	89	164
		2016/1/22	39	58
		2016/1/23	30	39
		2016/1/24	33	17
		2016/1/25	53	33
		2016/1/26	56	40
		2016/1/27	100	154
		2016/1/28	106	169
		2016/1/29	66	108
		2016/1/30	47	68
		2016/1/31	57	85
**Average concentrations**			**76.5**	**130.77**

Table S11. Concentrations monitored by air quality monitoring stations in Tangshan.

City	Air Quality Monitoring Station	Date	Monitored Concentrations (Unit: μg/m³)	
			NO_2	$PM_{2.5}$
Tangshan	1. Supply and marketing cooperatives<br>2. Ceramics Company<br>3. No. 12 middle school<br>4. Xiaoshan Station<br>5. Commodities Bureau<br>6. Radar station	2016/1/1	102	206
		2016/1/2	133	342
		2016/1/3	94	183
		2016/1/4	66	72
		2016/1/5	48	46
		2016/1/6	59	64
		2016/1/7	40	33
		2016/1/8	54	36
		2016/1/9	90	134
		2016/1/10	60	75
		2016/1/11	56	48
		2016/1/12	45	36
		2016/1/13	44	29
		2016/1/14	80	88
		2016/1/15	109	159
		2016/1/16	67	76
		2016/1/17	55	66
		2016/1/18	59	37

	2016/1/19	84	44
	2016/1/20	80	74
	2016/1/21	62	86
	2016/1/22	38	44
	2016/1/23	22	18
	2016/1/24	21	16
	2016/1/25	53	44
	2016/1/26	58	51
	2016/1/27	84	81
	2016/1/28	92	105
	2016/1/29	49	39
	2016/1/30	62	60
	2016/1/31	54	42
**Average concentrations**		**65.2**	**78.52**

Table S12. Concentrations monitored by air quality monitoring stations in Xingtai.

City	Air Quality Monitoring Station	Date	Monitored Concentrations (Unit: μg/m^3)	
			NO_2	$PM_{2.5}$
Xingtai	1. Dahuo fontain<br>2. Xingtai Normal College<br>3. Road and Bridge Corporation<br>4. Environment protection office	2016/1/1	142	239
		2016/1/2	180	405
		2016/1/3	172	362
		2016/1/4	85	140
		2016/1/5	70	74
		2016/1/6	95	128
		2016/1/7	78	120
		2016/1/8	94	142
		2016/1/9	99	217
		2016/1/10	112	275
		2016/1/11	50	50
		2016/1/12	72	76
		2016/1/13	72	78
		2016/1/14	74	76
		2016/1/15	86	102
		2016/1/16	100	166
		2016/1/17	64	108
		2016/1/18	67	102
		2016/1/19	85	114
		2016/1/20	98	150
		2016/1/21	97	142
		2016/1/22	57	70
		2016/1/23	32	42
		2016/1/24	46	42
		2016/1/25	66	56
		2016/1/26	68	56
		2016/1/27	96	113
		2016/1/28	112	130
		2016/1/29	71	100
		2016/1/30	36	44
		2016/1/31	59	77
**Average concentrations**			**85**	**128.9**

Table S13. Concentrations monitored by air quality monitoring stations in Zhangjiakou.

City	Air Quality Monitoring Station	Date	Monitored Concentrations (Unit: μg/m^3)	
			NO_2	$PM_{2.5}$
Zhangjiakou	1. People's Park<br>2. Probe factory<br>3. Century Compound<br>4. North Pumping Station	2016/1/1	56	70
		2016/1/2	36	44
		2016/1/3	26	29
		2016/1/4	15	16
		2016/1/5	19	18
		2016/1/6	20	22
		2016/1/7	20	19
		2016/1/8	22	20
		2016/1/9	33	27
		2016/1/10	18	22
		2016/1/11	12	15
		2016/1/12	20	20
		2016/1/13	20	17
		2016/1/14	24	21
		2016/1/15	51	45
		2016/1/16	64	95
		2016/1/17	10	19
		2016/1/18	12	17
		2016/1/19	29	24
		2016/1/20	33	29
		2016/1/21	16	23
		2016/1/22	7	14
		2016/1/23	6	16
		2016/1/24	15	16
		2016/1/25	26	23
		2016/1/26	30	26
		2016/1/27	42	40
		2016/1/28	27	28
		2016/1/29	45	48
		2016/1/30	14	16
		2016/1/31	17	17
**Average concentrations**			**25.3**	**27.61**

Table S14. Parameterization scheme for WRF.

Model Parameterization	WRF
Domain setting	D1: 1,320 km × 1,320 km; D2: 864 km × 960 km
Microphysical process scheme	WSM6
Longwave radiation scheme	New Goddard
Solar radiation scheme	RRTM
Surface layer scheme	Pleim Xiu
Boundary layer scheme	ACM2
Cumulus convection scheme	Kain–Fritsch

Table S15. Parameterization used in CALPUFF.

Model Parameterization	CALPUFF	Model Parameterization	CALPUFF
Model version	6.42	Vertical diffusion	Gaussian
Domain setting	576 km × 738 km	Chemical mechanism flag (MCHEM)	MESOPUFF II
Nested grids	Single layer grid	Plume rise	Briggs Plume Rise
Horizontal resolution	6 km	Terrain adjustment	Partial Plume Path Adjustment
Number of vertical layers	11	Building downwash	ISC method
Horizontal diffusion	Gaussian	Dry deposition	Vertical Structure and Mass Depletion/Resistance Deposition

Table S16. The transfer coefficient matrix of NO_x.

NO_x (Unit: $\times 10^{-3}$ μg/m^3•t)	Emission City												
Receptor	Beijing	Baoding	Handan	Liangfang	Cangzhou	Hengshui	Tianjin	Shijiazhuang	Qinhuangdao	Chengde	Tangshan	Xingtai	Zhangjiakou
Beijing	3.31	0.35	0	0.24	0	0.52	0	0.35	0.09	0	0.25	0	0.3
Baoding	0.72	4.08	0.04	0.08	0.23	0.28	0	1.46	0.17	0	0.99	0	0.03
Handan	0.14	0.14	3.64	0.3	1.24	0.01	0.09	0.54	0.01	0.41	0.01	0.32	0.01
Liangfang	0.71	0.24	0.13	3.6	0.22	0.03	0.01	0.89	0.03	0.19	0.07	0.02	0.05
Cangzhou	0.39	1.11	1.01	0.21	3.51	0.06	0.02	2.01	0.06	0.1	0.13	0.09	0.02
Hengshui	0.23	0.02	0	0	0	3.04	0	0.01	0.07	0	0.1	0	0.04
Tianjin	0.03	0.03	1.19	0.06	0.27	0	5.17	0.11	0	0.41	0	3.29	0
Shijiazhuang	1.93	1.86	0.14	0.37	0.41	0.17	0	4.66	0.11	0.02	0.43	0.01	0.08
Qinhuangdao	0.05	0.16	0	0.01	0.02	1.75	0	0.05	1.73	0	1.81	0	0.01
Chengde	0.09	0.05	0.31	1.15	0.16	0	0.05	0.22	0.01	3.27	0.01	0.2	0
Tangshan	0.17	0.81	0	0.02	0.03	1	0	0.24	0.39	0	4.1	0	0.01
Xingtai	0.08	0.05	2.46	0.23	0.54	0	1.03	0.27	0	1.45	0	4.81	0
Zhangjiakou	0.05	0	0	0.03	0	0	0	0	0	0	0	0	0.84
Sum	7.9	8.9	8.92	6.3	6.63	6.86	6.37	10.81	2.67	5.85	7.9	8.74	1.39

Table S17. The transfer coefficient matrix of $PM_{2.5}$.

$PM_{2.5}$ (Unit: ×10^{-3} μg/m^3•t)	Emission City												
Receptor	Qinhuangdao	Tianjin	Handan	Xingtai	Cangzhou	Chengde	Baoding	Langfang	Beijing	Hengshui	Tangshan	Shijiazhuang	Zhangjiakou
Qinhuangdao	24.67	14.62	8.94	18.41	9.28	8.53	4.35	23.08	5.22	8.56	9.38	5.95	4.13
Tianjin	5.18	36.63	10.11	8.79	15.62	2.17	5.18	20.88	3.41	7.12	12.43	6.54	1.58
Handan	1.8	4.06	56.23	9.32	16.09	1.15	22.33	6.12	0.65	12.98	1.5	32.02	1.84
Xingtai	9.77	8.26	14.13	57.19	9.25	3.11	6.72	15.03	3.41	30.98	5.61	10.73	6.23
Cangzhou	2.18	6.94	34.05	8.84	35.94	1.58	12.2	9.31	1.28	8.5	2.87	15.95	1.61
Chengde	7.56	8.2	2.63	3.89	3.5	20.79	1.51	6.02	23.12	1.99	23.16	1.83	1.67
Baoding	0.53	1.01	6.68	2.34	2.86	0.24	58.59	1.56	0.12	5.32	0.33	21.24	0.64
Langfang	6.15	20.3	19.72	21.33	25.08	2.57	8.93	44.87	3.09	14.58	7.16	12.08	2.35
Beijing	2.34	6.96	2.61	3.79	3.28	2.72	1.35	5.37	29.01	3.14	15.15	1.95	0.83
Hengshui	2.49	2.98	21.02	13.48	7.8	0.97	17.46	5.19	0.7	57.51	1.44	32.84	2.65
Tangshan	3.48	15.47	3.91	5.17	4.81	2.92	2.53	8.95	12.02	4.38	40.34	3.24	0.98
Shijiazhuang	0.91	1.52	15.71	4.43	5.35	0.5	49.74	2.35	0.29	11.7	0.63	59.02	1.14
Zhangjiakou	5.83	2.66	1.53	3.48	1.71	3.6	0.64	3.46	1.74	1.32	2.48	0.94	12.55
Sum	72.89	129.61	197.27	160.46	140.57	50.85	191.53	152.19	84.06	168.08	122.48	204.33	38.2

Table S18. The maximum allowable emissions and the emissions variation ratio of two pollutants under different emission scenarios.

(a) NO_x

Region	S0	S1	S1 Variation Ratio	S2	S2 Variation Ratio
ZJK	7.47	4.26	-0.43	9.79	0.31
CD	14.78	7.83	-0.47	18.77	0.27
QHD	3.48	2.09	-0.40	3.83	0.10
BJ	25.90	12.17	-0.53	26.94	0.04
CZ	5.81	3.31	-0.43	5.92	0.02
TJ	27.79	16.39	-0.41	25.84	-0.07
HS	8.06	3.38	-0.58	5.32	-0.34
XT	11.24	5.73	-0.49	7.31	-0.35
TS	30.87	23.15	-0.25	19.75	-0.36
LF	9.90	5.05	-0.49	5.25	-0.47
BD	17.37	8.51	-0.51	9.03	-0.48
HD	17.59	8.97	-0.49	8.97	-0.49
SJZ	23.64	13.48	-0.43	9.69	-0.59
Average variation ratios	15.68	8.79	-0.44	11.95	-0.23

(b) $PM_{2.5}$

Region	S0	S1	S1 Variation Ratio	S2	S2 Variation Ratio
ZJK	3.89	2.22	-0.43	7.12	0.83
QHD	2.00	1.16	-0.42	3.44	0.72
CD	7.48	4.34	-0.42	8.61	0.15
TJ	10.62	4.46	-0.58	6.48	-0.39
HD	8.83	4.50	-0.49	3.71	-0.58
XT	6.77	3.93	-0.42	2.78	-0.59
HS	4.74	2.42	-0.49	1.85	-0.61
TS	7.57	4.16	-0.45	2.95	-0.61
CZ	3.18	1.78	-0.44	1.21	-0.62
BJ	8.25	5.20	-0.37	2.64	-0.68
BD	10.33	4.85	-0.53	2.99	-0.71
LF	4.95	2.13	-0.57	1.39	-0.72
SJZ	9.88	5.04	-0.49	2.67	-0.73
Average variation ratios	6.81	3.55	-0.48	2.59	-0.46

Table S19. The GDP and the variation ratio of GDP under different emission scenarios.

Region	S0	S1	S1 Variation Ratio	S2	S2 Variation Ratio
BD	1.40	0.68	-0.51	0.69	-0.51
BJ	31.46	15.14	-0.52	31.12	-0.01
CD	0.37	0.20	-0.46	0.47	0.26
CZ	0.87	0.50	-0.43	0.82	-0.05
HD	1.67	0.85	-0.49	0.84	-0.50
HS	0.49	0.21	-0.57	0.31	-0.37
LF	1.11	0.56	-0.50	0.56	-0.50
QHD	1.14	0.68	-0.40	1.34	0.17
SJZ	3.94	2.22	-0.44	1.57	-0.60
TJ	21.92	12.62	-0.42	19.80	-0.10
TS	4.07	3.01	-0.26	2.55	-0.37
XT	0.39	0.20	-0.48	0.24	-0.38
ZJK	0.82	0.46	-0.43	1.11	0.37
Average variation ratios	5.36	2.87	-0.46	4.72	-0.12

Table S20. The variation ratio of GDP in three hypothetical cases.

Region	Unchanged Emission Intensity		Emission Intensity Reduced by 20%		Emission Intensity Reduced by 50%	
	S1	S2	S1’	S2’	S1’’	S2’’
ZJK	-0.43	0.37	-0.29	0.71	0.14	1.73
CD	-0.46	0.26	-0.33	0.57	0.07	1.51
QHD	-0.40	0.17	-0.25	0.47	0.20	1.35
BJ	-0.52	-0.01	-0.40	0.24	-0.04	0.98
CZ	-0.43	-0.05	-0.29	0.18	0.14	0.89
TJ	-0.42	-0.10	-0.28	0.13	0.15	0.81
HS	-0.57	-0.37	-0.46	-0.22	-0.14	0.25
TS	-0.26	-0.37	-0.08	-0.22	0.48	0.25
XT	-0.48	-0.38	-0.35	-0.23	0.04	0.24
LF	-0.50	-0.50	-0.37	-0.37	0.00	0.01
HD	-0.49	-0.50	-0.36	-0.37	0.02	0.00
BD	-0.51	-0.51	-0.39	-0.39	-0.02	-0.02
SJZ	-0.44	-0.60	-0.29	-0.50	0.13	-0.21
Average variation ratios	-0.46	0.12	-0.32	0.00	0.09	0.60

Table S21. Emission reduction ratio in scenario S1 (relative to 2016).

Unit: %

	BJ	TJ	SJZ	CD	ZJK	QHD	TS	LF	BD	CZ	HS	XT	HD
NO_x	53	25	43	43	43	40	41	49	51	47	58	49	49
$PM_{2.5}$	37	45	49	44	43	42	58	57	53	42	49	42	49

Note: The data come from information not yet made public by the government.

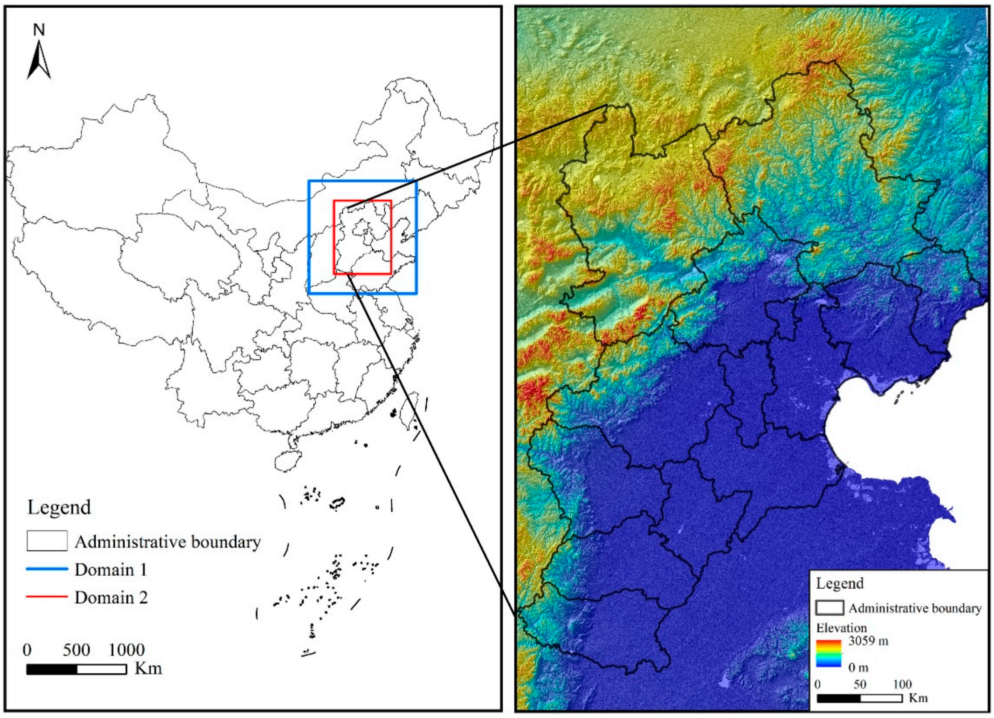

Figure S1. Domain coverage.
